# Interleukin 16 in lupus nephritis—a role for Th1 and CD8+ T cell migration

**DOI:** 10.1093/cei/uxaf068

**Published:** 2025-10-08

**Authors:** Kittikorn Wangriatisak, Francesca Faustini, Masa Filipovic, Heidi Wähämaa, Vivianne Malmström, Iva Gunnarsson, Vilija Oke

**Affiliations:** Division of Rheumatology, Department of Medicine Solna, Karolinska Institutet, Karolinska University Hospital, Stockholm, Sweden; Center for Molecular Medicine, Karolinska Institutet, Stockholm, Sweden; Division of Rheumatology, Department of Medicine Solna, Karolinska Institutet, Karolinska University Hospital, Stockholm, Sweden; Medicine Unit Dermatology, Gastroenterology, Rheumatology, Section of Rheumatology, Karolinska University Hospital, Stockholm, Sweden; Division of Rheumatology, Department of Medicine Solna, Karolinska Institutet, Karolinska University Hospital, Stockholm, Sweden; Center for Molecular Medicine, Karolinska Institutet, Stockholm, Sweden; Division of Rheumatology, Department of Medicine Solna, Karolinska Institutet, Karolinska University Hospital, Stockholm, Sweden; Center for Molecular Medicine, Karolinska Institutet, Stockholm, Sweden; Division of Rheumatology, Department of Medicine Solna, Karolinska Institutet, Karolinska University Hospital, Stockholm, Sweden; Center for Molecular Medicine, Karolinska Institutet, Stockholm, Sweden; Division of Rheumatology, Department of Medicine Solna, Karolinska Institutet, Karolinska University Hospital, Stockholm, Sweden; Medicine Unit Dermatology, Gastroenterology, Rheumatology, Section of Rheumatology, Karolinska University Hospital, Stockholm, Sweden; Division of Rheumatology, Department of Medicine Solna, Karolinska Institutet, Karolinska University Hospital, Stockholm, Sweden; Centre for Rheumatology, Academic Specialist Centre, Stockholm Region, Sweden

**Keywords:** interleukin 16, systemic lupus erythematosus, B cells, T cells, lupus nephritis

## Abstract

Dysregulation of interleukin (IL) 16 has been implicated in SLE, yet its cellular source and role in disease pathogenesis remain unclear. We analysed circulating IL16+ immune cells from 40 SLE patients, including 32 with active disease (SLEDAI-2K ≥ 4) using spectral flow cytometry. Plasma (pIL16) and urine IL16 (uIL16) levels were measured, and correlations with clinical variables were assessed. IL16 effects on T cell migration were studied *in vitro*. Active SLE patients showed broadly reduced proportions of cells expressing IL16, including CD4+T, CD8+T, B, and NK cells. This reduction was prominent in several cell subsets including Th1-like cells and plasmablasts. Further sub-analyses of lupus nephritis (LN) versus non-LN, demonstrated significantly reduced IL16 expression e.g., in Th1-like and double negative B cell subsets in LN. In parallel, SLE patients displayed increased pIL16 levels, and LN patients showed increased uIL16 which associated positively with disease activity SLEDAI-2K index and negatively with complement C4 levels and IL16+CD4+T-cell counts. *In vitro,* IL16 induced CXCR4 and CCR5 mediated migration of Th1-cells and attracted CD8+T cells via CXCR4, which was partially inhibited by IL16 blockade. We demonstrate reduced intracellular IL16 expression in SLE lymphocytes, with low IL16+CD4+T cell proportions in LN correlating with increased uIL16. Extracellular IL16 may drive Th1 and CD8+T cell infiltration, contributing to organ inflammation. IL16 blockade reduced T cell migration, highlighting its potential as therapeutic target.

## Teaser

Dysregulation of interleukin (IL) 16 has been implicated in SLE, yet its cellular source and role in disease pathogenesis remain unclear. We analysed circulating IL16+ immune cells from forty SLE patients, including 32 with active disease (SLEDAI-2K ≥ 4) using spectral flow cytometry. Plasma (pIL16) and urine IL16 (uIL16) levels were measured, and correlations with clinical variables were assessed. IL16 effects on T cell migration were studied *in vitro*. Active SLE patients showed broadly reduced proportions of cells expressing IL16, including CD4+T, CD8+T, B, and NK cells. We demonstrate reduced intracellular IL16 expression in SLE lymphocytes, with low IL16+CD4+T cell proportions in LN correlating with increased uIL16. Extracellular IL16 may drive Th1 and CD8+T cell infiltration, contributing to organ inflammation. IL16 blockade reduced T cell migration, highlighting its potential as therapeutic target. Graphical abstract created in BioRender. Wangriatisak, K. (2025) https://www.biorender.com/foa59mw

## Graphical Abstract

**Graphical Abstract:**
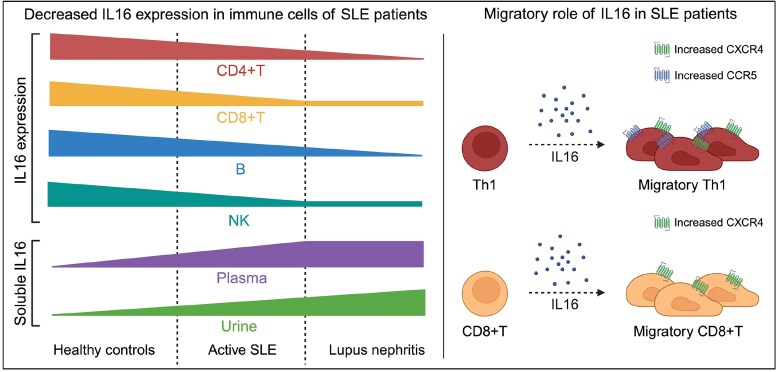

## Introduction

Systemic lupus erythematosus (SLE) is a heterogeneous autoimmune disease which presents with a complex array of symptoms and abnormalities in the immune system [1–4]. Autoantibodies (autoAbs) [5, 6], immune cells and cytokine dysregulation are relevant in lupus development [7, 8].

Interleukin (IL) 16, a homotetrameric protein, was described in 1982 as lymphocyte chemoattractant factor (LCF) due to its role in recruiting CD4+T cells [9]. In 1995, it was renamed IL16 given its broader functions such as the ability to modulate immune responses [10, 11]. Since then, it has been implicated in asthma and allergies, autoimmunity, and cancer [12, 13]. Patients with SLE show increased circulating IL16 [14, 15] which was also identified in lupus (LE) skin lesions and urine in lupus nephritis (LN) [14, 16, 17].

Immune cells of adaptive and innate origin can produce IL16 [18–21], which is stored in cytoplasm and needs to be cleaved by active caspase-3 to produce a secretory biologically active C-terminal fragment [22]. Active IL16 facilitates activation, chemoattraction, and migration of CD4+T cells and may interact with the chemokine receptors C-C chemokine receptor 5 (CCR5)-predominantly expressed on Th1 cells [23, 24]-and C-X-C chemokine receptor 4 (CXCR4) [25, 26].

Altogether, indirect evidence indicates that IL16 could contribute to SLE pathogenesis, but its cellular sources and detailed mechanisms remain poorly understood, including whether targeting IL16 could have therapeutic benefit.

In this study we mapped which circulating cell subsets express IL16 (IL16+) and measured soluble IL16 levels in plasma and urine; we explored associations of these measurements with clinical SLE parameters and investigated possible roles of IL16 in plasma cell differentiation and T cell migration. Further, we tested whether inhibition of IL16 could attenuate its effects on the functions of immune cells.

## Materials and methods

### Patients and samples

Patients were older than 18 years and met the 1982 American College of Rheumatology (ACR) and 2012 Systemic Lupus International Collaborating Clinics (SLICC) classification criteria for SLE [27, 28]. Patients received standard of care treatment. Collected data consisted of demographics, clinical manifestations, and laboratory parameters. Exclusion criteria were presence of overlapping syndrome, and ongoing or suspected cancer or infection.

In total, 40 SLE patients were recruited with inactive (I-SLE) (*n* = 8; SLEDAI-2K score < 4) and active disease (A-SLE) (*n* = 32; SLEDAI-2K score ≥ 4), of whom lupus nephritis (LN), *n* = 16 (Supplementary Figure S1). Blood samples were collected to identify IL16+ cell subsets. Blood samples from A-SLE group were used to investigate the effect of IL16 in inducing plasma cell differentiation (*n* = 3) and T cell migration (*n* = 7). Healthy donors (HC, *n* = 15) matched by gender and age served as control comparators in assessment of circulating IL16+ cells and in *in vitro* experiments (*n* = 7).

### Routine laboratory parameters

Complement levels (C1q, C3, C3d, and C4) were measured by nephelometry at the Department of Clinical Chemistry, Karolinska University Hospital (KS). Renal function was assessed by plasma creatinine measurements (µmol/L) and estimated glomerular filtration rate (eGFR) was calculated using Malmö equation [29]. Autoantibodies (anti-dsDNA) were analysed by indirect immunofluorescence method (IFL) at the Department of Clinical Immunology, KS.

### Identification of IL16-positive cells by spectral flow cytometry

**Figure 1.**
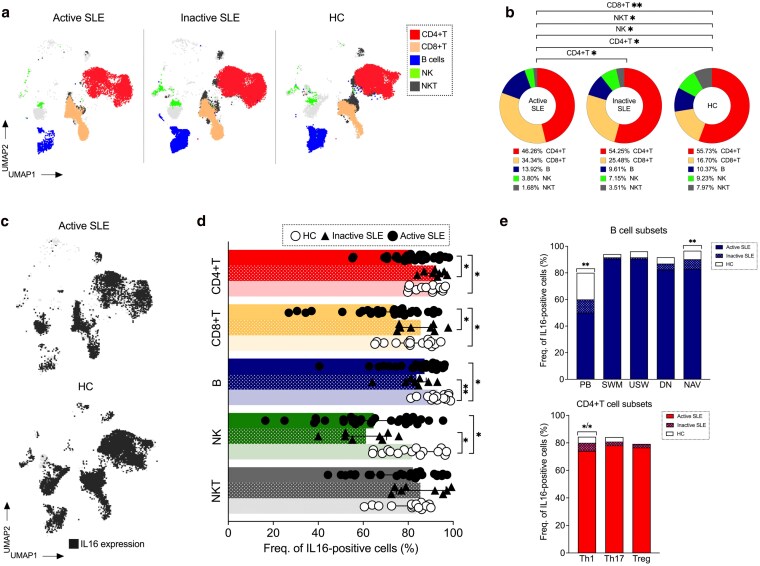
Reduced IL16 expression in immune cells of active SLE patients. (**a**) Overlay of uniform manifold approximation and projections (UMAPs) of lymphocyte subpopulations (*n* = 50 000 events) from patients with active, inactive SLE and HC. (**b**) Bar charts showing the proportion of T (CD4 and CD8), B, natural killer (NK) and natural killer T (NKT) cells in patients with active (*n* = 32), inactive (*n* = 8), and HC (*n* = 15). (**c**) UMAPs representing IL16+ cells (black dots) in active SLE and HC. Frequencies of IL16+ cells in (**d**) major immune compartments (CD4+T, CD8+T, B, NK, and NKT) and (**e**) B- and T-cell subsets among patients (active and inactive) and HC groups. Data in D and E were represented as the median and interquartile range. Comparison of cell frequencies among different groups was analysed by Kruskal–Wallis test and *P*-values were corrected by Dunn’s test for multiple comparisons. Only statistically significant *P*-values < 0.05 are presented. PB, plasmablast; SWM, switched memory; USW, unswitched memory; DN, double negative; NAV, naive; Th, T helper; Treg, T regulatory

Peripheral blood mononuclear cells (PBMCs) from patients (I-SLE, *n* = 8 and A-SLE, *n* = 32) were surface stained with fluorochrome-labelled antibodies (Abs) and viability dye according to manufacturer’s instructions (Supplementary Table S1). After fixation and permeabilization, cells were intracellularly stained with Abs against IL16. FACS data were acquired with a 5-laser spectral flow cytometer and analysed using Flowjo V10.10.0 Software. Positive IL16 staining was assessed in different immune cell compartments including CD4+T (CD3^+^CD8^−^CD4^+^), CD8+T (CD3^+^CD4^−^CD8^+^), B (CD3^−^CD19^+^), natural killer (NK, CD3^−^CD56^+^), and natural killer T (NKT, CD3^+^CD56^+^) cells (Fig. 1 and Supplementary Figure S2A). IL16 expression was also investigated in plasmablasts (PB: CD27^hi^CD38^hi^), switched memory (SWM: CD27^+^IgD^−^), unswitched memory (USW: CD27^+^IgD^+^), double negative (DN: CD27^−^IgD^−^), and naive (NAV: CD27^−^IgD^+^) (Supplementary Figure S2B); and T helper 1-like (CXCR3^+^CCR6^−^), T helper 17-like (CXCR3^−^CCR6^+^), non-Th1/Th17-like (CXCR3^−^CCR6^−^), and regulatory T (Treg: CD127^−^CD25^+^) cells (Supplementary Figure S2C).

### Public dataset and online analysis tool

A publicly available single cell RNA-sequencing dataset from the Accelerating Medicines Partnership (AMP) project, was accessed through the Broad Institute’s Single Cell Portal. IL16 expression was investigated in the twenty-two reported clusters, including 21 immune cells and one epithelial cell cluster [30].

### IL16 measurements

Plasma (pIL16) and urine (uIL16) levels were measured by a commercial ELISA kit (R&D, DuoSet, Cat: DY316) according to manufacturer’s instructions. The detection cut-off was 10 pg/ml. pIL16 was measured in 28 (LN, *n* = 13 and non-LN, *n* = 15), and uIL16 in 19 (LN, *n* = 10 and non-LN, *n* = 9) patients.

### Plasma cell differentiation

PBMCs (2 × 10^5^ cells) from A-SLE patients (*n* = 3) were cultured in RPMI media (RPMI with 10% fetal bovine serum, 2 mM L-glutamine, 100 U/ml penicillin, 100 µg/ml streptomycin, and 10 mM HEPES) supplemented with one of four stimuli: stimuli 1 (S1), 1 µg/ml R848 (Sigma), and 10 ng/ml IL2 (PeproTech); stimuli 2 (S2), 1 µg/ml R848, 10 ng/ml IL2, and 50 ng/ml IL16 (Biotechne); stimuli 3 (S3), 1 µg/ml R848, 10 ng/ml IL2, and 50 ng/ml IL21 (PeproTech); stimuli 4 (S4), 1 µg/ml R848, 10 ng/ml IL2, 50 ng/ml IL16, and 50 ng/ml IL21. After culture at 37°C, 5% CO_2_ for 5 days, harvested cells were analysed for plasma cell differentiation and immunoglobulin were quantified in culture supernatants.

### IgG and IgM measurements

IgG and IgM antibody levels were measured by ELISA. Briefly, 2 µg/ml anti-human IgG (Jacksonimmuno) or 3 µg/ml anti-human IgM (Jacksonimmuno) was coated on 96-well plates. After blocking, diluted supernatants (1:10) were added to wells followed by goat anti-human IgG or IgM conjugated to horseradish peroxidase (HRP) (Jacksonimmuno). Plates were developed with tetramethylbenzidine enzyme substrate (TMB; Sigma) and absorbance was measured at 450 nm.

### T-cell sorting

PBMCs were stained with fluorochrome-labelled antibodies as reported in Supplementary Table S2. To explore Th cell migratory capacity, CD4+T cells from SLE patients (*n* = 4) and HC (*n* = 4) were sorted on SH800s Cell Sorter (USA). The purity of sorted CD4+T cells was >95%.

### Chemotaxis assays

*In vitro* chemotaxis assays were performed as previously described [23]. Cell migration was assessed using 96-well HTS transwell plates with an 8 µm pore size (Corning). Briefly, PBMCs (500 000 cells/well) from three A-SLE patients or sorted CD4+T cells (60 000 cells/well) from four A-SLE patients were stimulated with increasing concentrations of IL16: 10, 10^2^, 10^3^, and 10^4^ pg/ml. After 4 h incubation at 37°C with 5% CO_2_, cells migrated to the lower chamber were harvested and stained with monoclonal antibodies. The migrating cells were stained with monoclonal antibodies. RANTES (CCL5; 50 ng/ml) and unstimulated cells (US) were used as a positive control and negative control, respectively.

To inhibit IL16-mediated migration, we first incubated PBMCs or sorted CD4+T cells with 10 µg/ml of anti-human IL16 (Biotechne) at 37°C for 30 mins. Then the cells were transferred to transwell plate and proceeded as mentioned above. The stimulated cells from lower chamber were acquired on a 5-laser spectral flow cytometer.

### Statistical analysis

Data was analysed using GraphPad Prism 9.5.0 software (GraphPad Software Inc.) and is represented as median and quartiles (M, Q1-Q3). Medians were compared by Mann–Whitney U test (two groups; unpaired variables), Wilcoxon signed rank tests (two groups; paired variables) or Kruskal–Wallis test (>2 groups) followed by Dunn’s multiple comparison test. Spearman’s test was applied to investigate correlations. *P-*values < 0.05 were considered statistically significant.

## Results

### Patient characteristics

The SLE patients were mostly females (90%, *n* = 36), median age was 41.5 (30.5–49.5) and SLEDAI-2K score 6 (4–12). Details are provided in Table 1. Supplementary Table S3 provides detailed characteristics of patients presented.

**Table 1.** Characteristics of systemic lupus erythematosus patients and healthy controls

Categories	Features	SLE		HC
		Active	Inactive	
**Demographic**	No. of patients, No (%)	32/40 (80%)	8/40 (20%)	15
	Age, years (#)	41.5 (30.5–49.5)	38.5 (31–52)	35 (30–58)
	Gender			
	Male, No (%) Female, No (%)	2/32 (6%) 30/32 (94%)	2/8 (25%) 6/8 (75%)	1/15 (7%) 14/15 (93%)
	SLEDAI-2K score	8 (6–12)	0 (0–1.5)	ND
	Disease duration, years (#)	13.5 (4–22)	4 (2–12)	ND
**Laboratory parameters**	Plasma creatinine, µmol/L (#)	67 (54.5–77.5)	76.5 (60–89)	ND
	ESR rates, mm/h (#)	24 (12.5–50)	17.5 (10.5–31.5)	ND
	C1q levels, mg/L (#)	144 (96–205)	126.5 (53.5–222)	ND
	C3 levels, g/L (#)	0.8 (0.62–0.96)	0.98 (0.9–1.13)	ND
	C3d levels, g/L (#)	8 (6.5–9)	8 (5–11)	ND
	C4 levels, g/L (#)	0.1 (0.08–0.17)	0.17 (0.14–0.22)	ND
	Anti-dsDNA positive, N0 (#)	19/32 (59%)	2/8 (25%)	ND
**Treatment at inclusion**	Mean dose (Prednisolone equivalent, mg)	7.5	5	ND
	Antimalarial	23/32 (72%)	6/8 (75%)	ND
***sDMARDs***	Azathioprine	0/32 (0%)	1/8 (12%)	ND
	Mycophenolate mofetil	8/32 (25%)	1/8 (12%)	ND
	Calcineurin inhibitors	2/32 (6%)	0/8 (0%)	ND
***bDMARDs***	Belimumab	0/32 (0%)	0/8 (0%)	ND
	Rituximab	4/32 (12%)	5/8 (62%)	ND

ND, not done; SLE, systemic lupus erythematosus; SLEDAI-2K, systemic lupus erythematosus disease activity 2000; ESR, erythrocyte sedimentation rate; C1q, complement 1q; C3, complement 3; C3d, complement 3d; C4, complement 4; Anti-dsDNA, anti-double strand DNA; sDMARDs, synthetic disease-modifying antirheumatic drugs; bDMARDs, biologic disease-modifying antirheumatic drugs; # Median, # Median (Q1–Q3).

### Distribution of peripheral blood lymphocyte subsets

We assessed lymphocyte subsets across groups (Fig. 1 and Supplementary Figure S2). Similar to other SLE cohorts, A-SLE patients showed decreased frequency of CD4+T (*P* = 0.03), NK (*P* = 0.02), and NKT (*P* = 0.02) cells, paralleled by an increased proportion of CD8+T (*P* = 0.002) cells, as compared with HC (Fig. 1b). The proportion of CD4+T cells was lower in A-SLE as compared to those with I-SLE (*P* = 0.04).

### Decreased IL16 expression in B and T cell subsets of active SLE patients

Intracellular IL16 expression has been reported in various cell subsets in health and disease. In our SLE samples IL16 could be detected in all lymphocyte subsets, but in different magnitude (Fig. 1c and Supplementary Figure S3). This finding was validated in a publicly available single-cell dataset [30], which profiles gene expression at single-cell level e.g. in B, T, and NK cells and even macrophages in kidney biopsies from LN patients (Supplementary Figure S4).

In A-SLE, the proportions of IL16+ CD4+T (*P* = 0.02), CD8 + T (*P* = 0.03), B (*P* = 0.01) and NK (*P* = 0.02) cells were reduced as compared to HC. Lower frequencies of IL16+ CD4+T (*P* = 0.02) and CD8+T cells (*P* = 0.02) were observed in A-SLE in comparison to I-SLE (Fig. 1d and Supplementary Figure S3B). Additionally, I-SLE exhibited lower percentage of IL16+ B (*P* = 0.005) and NK (*P* = 0.01) cells, as compared to HC (Fig. 1d).

We investigated IL16 expression in B and CD4+T cell subsets. We observed reduced frequency of IL16+ plasmablasts (PB, *P* = 0.005) and naive (NAV, *P* = 0.008) B cells in the A-SLE group, as compared to HC, with decreased IL16 expression primarily found in activated naive (aNAV) B cells (Supplementary Figure S5A). Similarly, the frequency of IL16+ T helper 1-like cells (Th1-like) was also decreased in A-SLE compared to HC (*P* = 0.02) and I-SLE patients (*P* = 0.04) (Fig. 1e).

### Decreased proportions of IL16+ B and CD4+T cell subsets in LN patients

**Figure 2.**
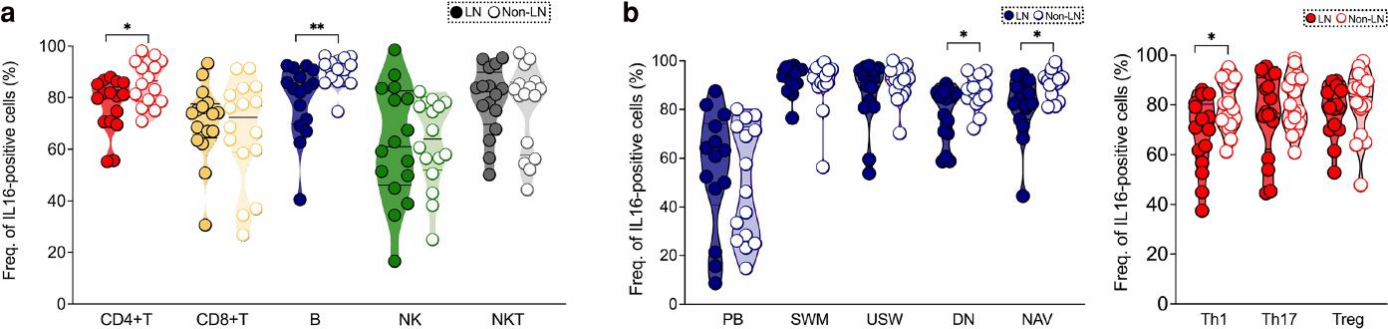
Deep mapping of IL16+ PBMCs in active SLE patients with and without lupus nephritis. (**a**) Frequencies of IL16+ cells in major immune cell types (CD4+T, CD8+T, B, NK and NKT) and (**b**) IL16+ B- and T-cell subsets among patients with LN (*n* = 16) and non-LN (*n* = 16). Dark and light dots represent LN and non-LN patients, respectively. Data in A and B is represented as the median and interquartile range and was analysed by Mann–Whitney U test. Only statistically significant *P*-values < 0.05 are presented. PB, plasmablast; SWM, switched memory; USW, unswitched memory; DN, double negative; NAV, naive; Th, T helper; Treg, T regulatory

We next explored IL16+ cell subsets in patients with LN in comparison to active non-LN. LN patients showed decreased frequency of IL16+ CD4+T (*P* = 0.02) and B (*P* = 0.002) cells (Fig. 2a). The lowest levels, indicative of highest release, was observed in NK cells but without a striking difference between LN and non-LN groups. Further analysis of B and CD4+T cell subsets revealed that reduced IL16 expression in LN patients was specifically observed in Th1-like (*P* = 0.03), double negative (DN, *P* = 0.02) and NAV B (*P* = 0.02) cells (Fig. 2b). Decreased IL16 expression was also found in LN aNAV, but the difference was not statistically significant (Supplementary Figure S5B).

### Soluble IL16 levels in plasma and urine of SLE and LN patients

**Figure 3.**
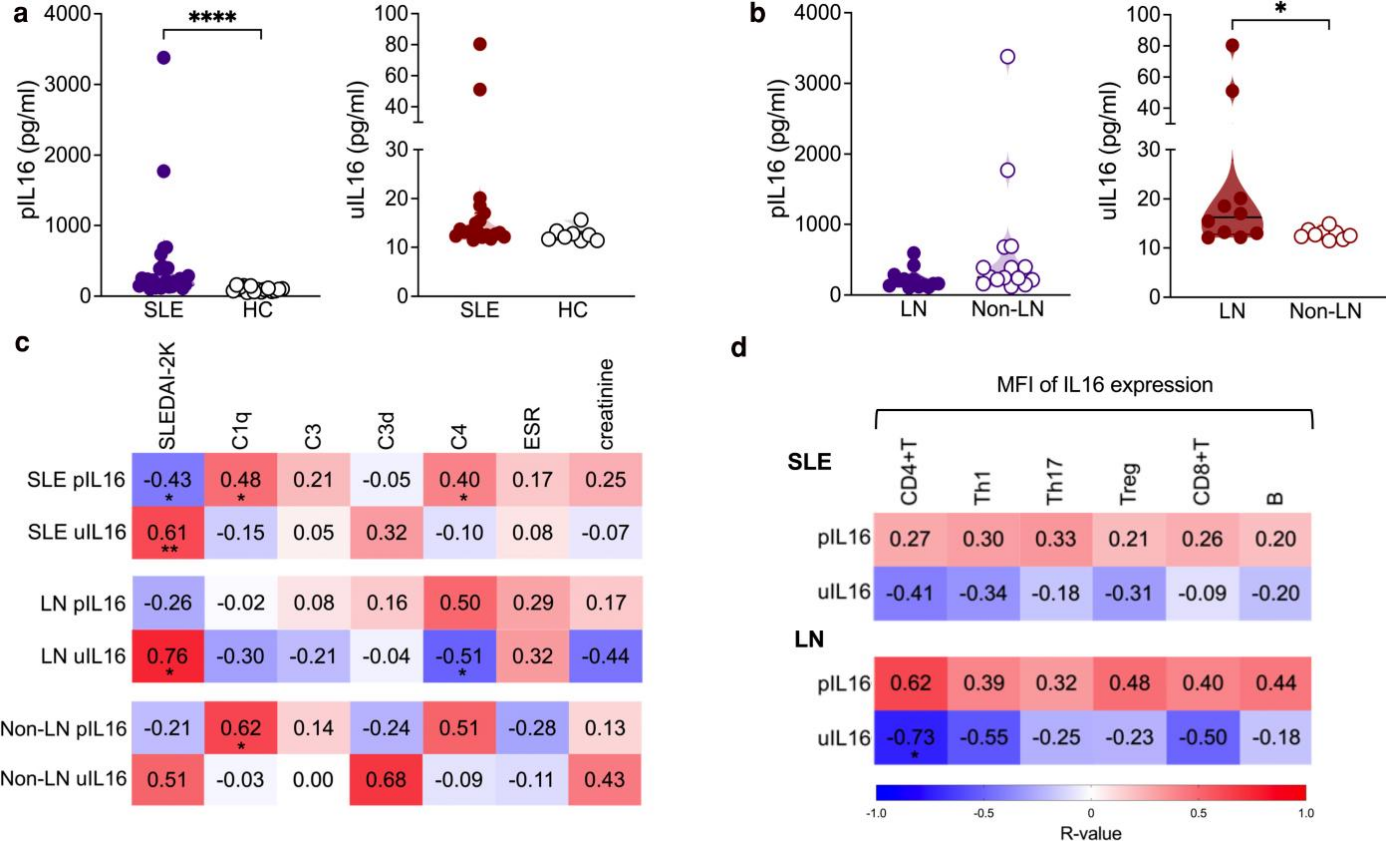
Associations among soluble IL16 levels, SLE disease activity measures and numbers of IL16+ PBMCs. (**a**) Levels of soluble IL16 in plasma (p) and urine (u) in SLE (plasma, *n* = 28 and urine, *n* = 19) and HC (plasma, *n* = 18 and urine, *n* = 8) and (**b**) in LN (plasma, *n* = 13 and urine, *n* = 10) and non-LN (plasma, *n* = 15 and urine, *n* = 9) patients. (**c**) Correlation matrix heatmap shoes associations with pIL16 and uIL16 levels and clinical variables of SLE in LN and non-LN patients, and (**d**) with expression levels of IL16 (median fluorescence intensity, MFI) in CD4+T, and their subsets (Th1, Th17, and Treg), CD8 + T and B cells in patients with SLE and LN. The colour intensity reflects the strength of the correlation (−1 to 1). Data in A and B were represented as the median and interquartile range. Baseline data of pIL16 and uIL16 levels were analysed by Mann–Whitney U test. Data in C and D, the correlation analysis was done using Spearman’s Rank coefficient (*r*). Only statistically significant *P*-values < 0.05 are presented

Levels of IL16 were measured in plasma (pIL16) and urine (uIL16) (Supplementary Table S4). Overall, SLE patients had higher pIL16 in comparison with HC (*P* < 0.0001) (Fig. 3a), but patients with high pIL16 had lower SLEDAI-2K scores than those with low pIL16. Conversely, in LN patients we found increased uIL16, which correlated negatively with dropping pIL16 (Fig. 3b and Supplementary Figure S6).

In a fraction of patients (plasma: LN, *n* = 5 and non-LN, *n* = 5 and urine: LN, *n* = 5 and non-LN, *n* = 2), we could assess longitudinal samples following treatment initiation (baseline, 3 and 6 months from LN diagnosis). Neither pIL16 nor uIL16 showed significant changes over time in this small subgroup (Supplementary Figure S6B).

### Correlations of soluble IL16 with clinical variables and IL16 expressing cells

We next investigated the association of pIL16 and uIL16 with clinical variables. We found a positive correlation between uIL16 and SLEDAI-2K (*r* = 0.61, *P* = 0.01) (Fig. 3c). In the LN group associations were more prominent, with uIL16 showing even stronger positive correlation with SLEDAI-2K score (*r* = 0.76, *P* = 0.03) and notable negative correlation with C4 levels (*r* = −0.51, *P* = 0.04) (Fig. 3c).

Since active patients showed lower proportions of IL16+ B and T cells (both CD4+ and CD8+), we considered the possibility that these cells could be a source of extracellular soluble IL16. Hence, we run a correlation analysis of levels of soluble IL16 and its intracellular expression from different cells. In LN, we found a negative correlation between uIL16 levels and IL16 expression in CD4+T cells (*r* = −0.73, *P* = 0.03) (Fig. 3d). Although not statistically significant, similar trend was noted for CD4+T cell subsets (Th1 and Th17-like) and CD8+T cells.

### IL16 did not affect plasma cell differentiation

Since we found reduced intracellular IL16 levels in plasmablasts, we attempted to clarify whether IL16 could affect plasma cell differentiation. To test this, we set up PBMC cultures of SLE patients and HC (Supplementary Table S5) with different conditions: S1 (R848 and IL2), S2 (R848, IL2, and IL16), S3 (R848, IL2, and IL21), and S4 (R848, IL2, IL16, and IL21) (Supplementary Figure S7A).

Adding IL16 did not exert any enhancing effect on plasma cell generation. A slightly higher plasma cell frequency was observed in presence of IL21 in SLE-derived cell cultures (significant only for condition S3), compared with HC: S3 (*P* = 0.04) and S4 (*P* = 0.07) (Supplementary Figure S7B). This result was reinforced by increased IgG levels from S3 (*P* = 0.04) and S4 (*P* = 0.048) in SLE patients, compared to HC. No difference in IgM levels was observed (Supplementary Figure S7C).

We also investigated whether IL16 could influence the activation status of plasma cells by examining HLA-DR and CD95 expression under different conditions (Supplementary Figure S7D). Neither HLA-DR nor CD95 expression was affected, indicating that adding IL16 to the culture milieu did not enhance activation of plasma cells. Compared to HC, HLA-DR upregulation of SLE plasma cells was observed in all conditions (Supplementary Figure S7E), which suggests that plasma cells of SLE patients may be more prone to activation and prompt to produce autoAbs independently of IL16.

### IL16 influences lymphocyte migration in a dose-dependent manner

**Figure 4.**
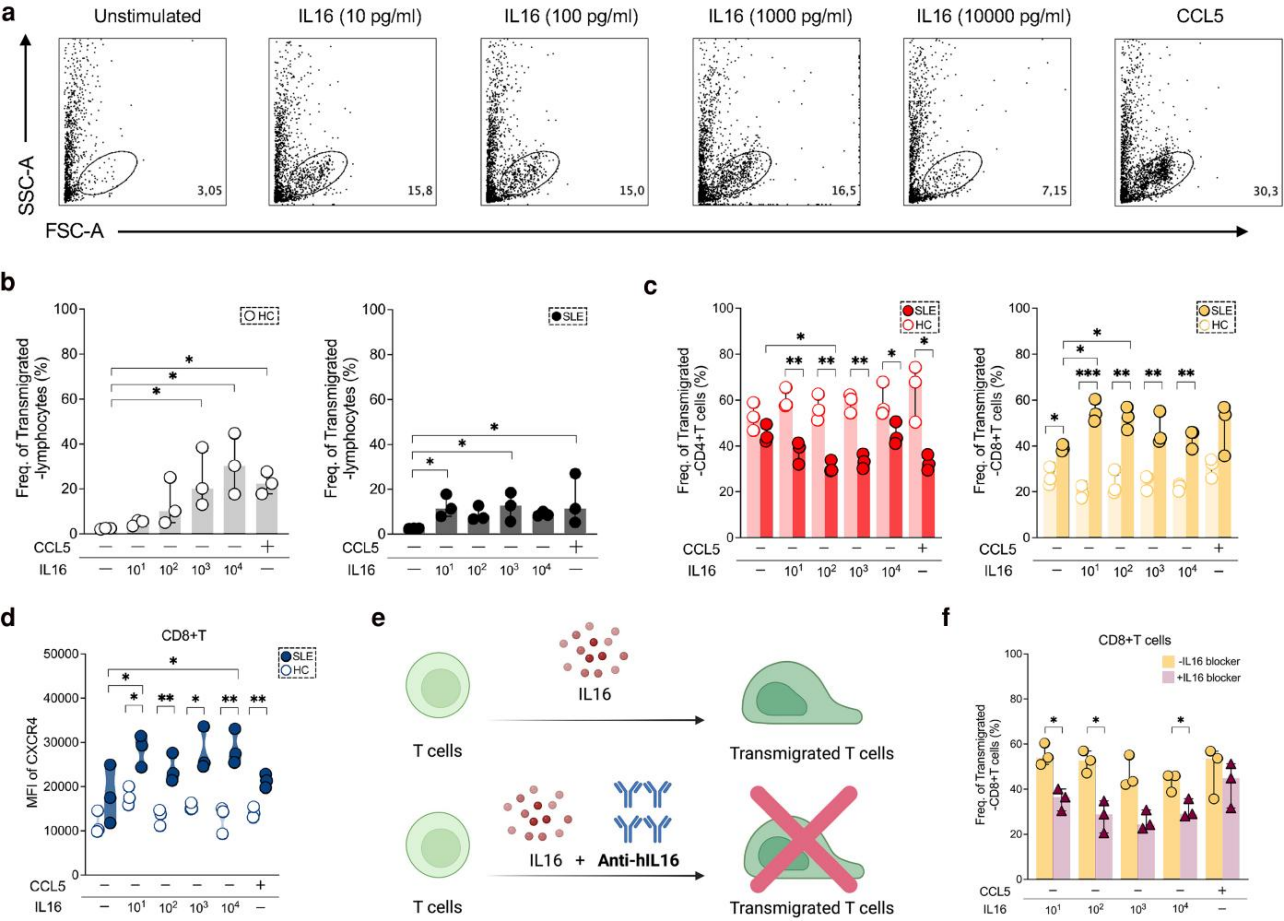
IL16 preferentially induces T cell migration in SLE. (**a**) Flow cytometric plots representing transmigrated lymphocytes of one A-SLE patients after IL16 (at different concentrations: 10, 10^2^, 10^3^, 10^4^ pg/ml and without stimulation) and CCL5 stimulation. Frequencies of migratory lymphocytes in (**b**) HC (left panel) and SLE (right panel), and of (**c**) CD4+T (left panel) and CD8+T cells (right panel) among HC (*n* = 3) and A-SLE (*n* = 3) after stimulation of CCL5 and IL16 (at 10, 10^2^, 10^3^, 10^4^ pg/ml), and unstimulated condition. (**d**) MFI of CXCR4 expression in migratory CD8+T cells under different stimulation conditions. (**e**) Cartoon depicting that IL16 induced T cell migration may be inhibited by addition of anti-human IL16. (**f**) Frequency of lupus transmigrated-CD8+ T cells after addition of anti-human IL16 antibodies to IL16 stimulated samples (at concentrations of 10, 10^2^, 10^3^, 10^4^ pg/ml). Data in B–D and F is represented as the median and interquartile range. All comparisons were analysed by Kruskal–Wallis test and *P* values were corrected by Dunn’s test for multiple comparisons. Group comparisons were analysed by Mann–Whitney U test. Only statistically significant *P* values < 0.05 are presented. Figure E Created in BioRender. Wangriatisak, K. (2025) https://BioRender.com/d7q4l5u

IL16 is known as a chemoattractant. Therefore, we investigated the effect of IL16 on lymphocyte migration using a chemotaxis assay (Fig. 4a) (Supplementary Table S5). In HC, IL16 stimulation induced dose-dependent lymphocyte migration, with significant peaks at concentrations of 10^3^ (*P* = 0.04) and 10^4^ pg/ml (*P* = 0.02) and of a similar magnitude as observed in positive control CCL5, in comparison to unstimulated (US) conditions. In SLE-derived PBMCs a response was also observed, albeit to a lower degree compared to HC cells, but of a similar magnitude as observed for positive control CCL5 (Fig. 4b).

We next compared the responses between patients and HC PBMCs. SLE patients showed an increased number of transmigrating lymphocytes already at 10 pg/ml (*P* = 0.03), with less response at the highest IL16 concentration (10^4^ pg/ml: *P* = 0.04) (Supplementary Figure S8A).

### IL16 skews T cell migration between SLE and controls

Next, we performed a sub-analysis of the effect of IL16 on migration of CD4+ and CD8+T cells. Addition of IL16 induced transmigration of CD4+T cells derived from controls, but SLE-derived CD4+T cells showed a lower response (Fig. 4c). Instead, stimulation with IL16 increased transmigration of patient derived CD8+T cells at all IL16 concentrations (Fig. 4c). This was not the case for HC CD8+T cells.

We next examined the expression levels of CXCR4 and CCR5 on migrating CD8+T cells. Surface staining revealed that stimulation under all tested conditions increased CXCR4-positive CD8+T proportions in SLE patients, but not in HC (Fig. 4d). This effect was not observed for CCR5 expression (Supplementary Figure S8B).

We sought to confirm this observation by using anti-human IL16 (anti-hIL16) in our experimental setting (Fig. 4e). Addition of anti-hIL16 to patient-derived PBMCs reduced the proportion of transmigrated-CD8+T cells at IL16 concentrations of 10 (*P* = 0.02), 10^2^ (*P* = 0.03) and 10^4^ (*P* = 0.03) pg/ml, as compared to conditions without blocker (Fig. 4f and Supplementary Figure S8C).

### IL16 induced th1-like cell migration in SLE

**Figure 5.**
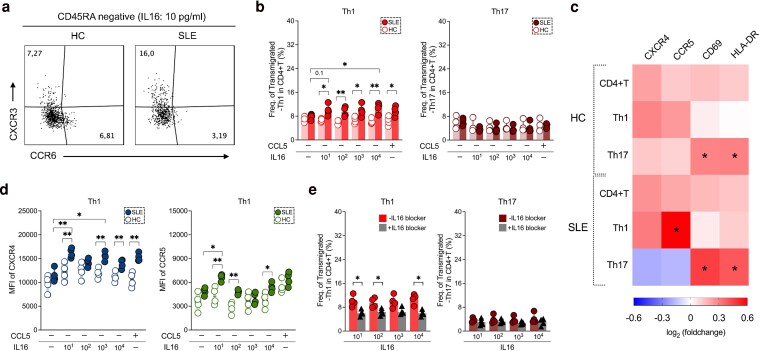
In SLE IL16 mediates Th1 cell migration via CCR5 which can be partially inhibited by anti-IL16 antibodies. (**a**) Flow cytometric plots representing transmigrated-Th1-like (CXCR3 + CCR6−) and Th17-like (CXCR3-CCR6+) of CD45RA-negative CD4+T cells in HC and A-SLE patient after 10 pg/ml of IL16 stimulation. (**b**) Frequencies of migratory-CD4+T cell subsets (Th1 and Th17-like) in A-SLE (*n* = 4) and HC (*n* = 4) after IL16 (at 10, 10^2^, 10^3^, 10^4^ pg/ml) and CCL5 stimulation. (**c**) Heatmap showing fold changes in MFI of surface CXCR4, CCR5, CD69 and HLA-DR expression on transmigrated cells relative to non-transmigrated cells. The colour intensity reflects the strength of the fold changes (−1 to 1). (**d**) MFI of CXCR4 (left panel) and CCR5 (right panel) expression in migratory Th1-like cells under different stimulation conditions. (**e**) Frequency of lupus transmigrated-Th1 and -Th17-like cells after addition of anti-human IL16 in different stimulated-IL16 concentrations (at 10, 10^2^, 10^3^, 10^4^ pg/ml). Data in B, D, and E are presented as the median and interquartile range and were analysed by Mann–Whitney U test. All comparisons were analysed by Kruskal–Wallis test and *P*-values were represented by Dunn’s multiple comparison test. Only statistically significant *P* values < 0.05 are presented

We next performed sub-analysis of transmigrated CD4 T cells. Analysing surface CXCR3 and CCR6 expression on memory CD4+T cells (CD45RA-CD4+), we focused on Th1-like (CXCR3 + CCR6) and Th17-like (CXCR3-CCR6+) subsets as they are implicated in SLE (Fig. 5a).

We purified CD4+T cells and under different stimulating conditions determined the percentage of migrating cells. Exposure to increasing IL16 concentrations induced migration of patient-, but not HC-derived Th1-like cells (IL16: 10, *P* = 0.01; 10^2^, *P* = 0.005; 10^3^, *P* = 0.03; 10^4^, *P* = 0.003 and CCL5: *P* = 0.03). Under these conditions we did not observe migration of the Th17-like subset (Fig. 5b), but both patient and HC-derived transmigrated Th17-like cells upregulated activation markers CD69 and HLA-DR (Fig. 5c).

### Migration of CCR5+ lupus th1-like cells is partially inhibited by IL16 blockade

We next explored which receptors could be involved in IL16 mediated Th1-cell migration. We examined expression levels of CXCR4, CCR5, and activation markers CD69 and HLA-DR on migrated cells (Fig. 5c). We found that SLE-derived migrated-Th1-like cells increased expression of both CXCR4 and CCR5 under IL16 stimulation at increasing concentrations (10, *P* = 0.009; 10^3^, *P* = 0.002; 10^4^, *P* = 0.008 for CXCR4 and CCL5, *P* = 0.002; respective values for CCR5 were 10, *P* = 0.007; 10^2^, *P* = 0.002 and 10^4^, *P* = 0.02) (Fig. 5d). Compared with US, IL16 at dose of 10 pg/ml induced significant upregulation of both CXCR4 (*P* = 0.002) and CCR5 (*P* = 0.04) on Th1-like cells, while IL16 at 10^3^ pg/ml could only enhance CXCR4 expression (*P* = 0.03) in SLE patients (Fig. 5d). Addition of anti-hIL16 reduced the proportion of migrating lupus Th1-like cells but had no impact on Th17-like cell transmigration (Fig. 5e and Supplementary Figure S8).

## Discussion

In this study we investigated the expression and function of IL16 in patients with active SLE, with a special focus on LN. The study idea was based on earlier reports implicating IL16 in SLE [14, 16].

The novelty of our study is the detailed mapping of circulating immune cell types expressing IL16 in patients and controls showing that IL16 is broadly expressed across subsets. Overall, many cell subsets in SLE were characterized by IL16 release, i.e. reduced intracellular expression. Here NK cells had the lowest IL16 content. Moreover, when comparing active and inactive patients, A-SLE IL16 levels were especially decreased in CD4+, CD4+Th1, CD8+T cells and plasmablasts. LN patients had the lowest levels of IL16+ CD4+T cells. The Th1-like subset was the most significantly affected, a pattern that was also observed, albeit to a lesser degree, in inactive patients. In addition, both active and inactive patients had low frequencies of circulating IL16+ B and NK cells compared to HC, with especially low proportions of IL16+ plasmablasts in active patients. LN patients showed even further reduction in IL16+ B cells. Detailed analysis revealed that the naive and double negative IL16+ B cells, with the related phenotypes activated naive and double negative 3 B cells, were the most affected. Intriguingly, this feature was particularly evident in expanded B cell subsets in SLE [31, 32]. These findings indicate that low levels of IL16+ B cells are a constitutive feature of SLE and associate with disease activity, e.g. in LN.

Increased levels of soluble IL16 in SLE patients was reported before [14, 15], and our data reveal that many cell types contribute to the extracellular IL16 which is readily detected in SLE. Somewhat contra-intuitively, lower disease activity was associated with higher pIL16 levels. Hence, patients with LN or high SLEDAI-2K displayed the lowest pIL16 levels. This suggests that pIL16 is not a reliable biomarker of disease activity but may indicate consumption or redistribution of IL16, for example, into target tissues in the severely ill patients. We and others reported that LN patients have increased uIL16 levels [14, 33]. We extend earlier reports by demonstrating a positive correlation of increasing uIL16 levels in LN patients with disease activity, but negatively with pIL16. In LN, increased uIL16 inversely correlates with decreased expression of IL16 in CD4+T cells, and a similar trend was observed for CD8+T cells. Decreased intracellular expression and dropping plasma IL16 levels could reflect its accumulation in inflamed tissues and release in urine in LN.

Aside from exploring the IL16 expressing peripheral cells and their relation with the concentration of the cytokine in plasma and urine, we explored effects of IL16 on immune cells. Regarding possible effects of soluble IL16 on lymphocyte function, we observed that *in vitro* exposure to IL16 facilitated migration, but that SLE-derived cells were less responsive than HC, possibly due to previous *in vivo* IL16 encounters. IL16 stimulation induced migration of both CD4+ and CD8+T cells, which is consistent with previous reports [26]. However, our study reveals that cell recruitment is diverging between SLE patients and HC, with preferential CD8+T cell migration in SLE via CXCR4. Interestingly, others have reported that CD8+T cells (with deficient cytotoxic capacity) represent the predominant T-cell subset among kidney-infiltrating cells in LN (class III or IV) and that CD8+T abundance correlates with disease activity [34–37]. We found that LN patients with elevated uIL16 levels had higher disease activity, which associated with loss of IL16 expression in CD8+T cells. These findings are in line with previous data on CD8+T cells containing cleaved bioactive IL16, which can be rapidly secreted upon stimulation [19]. Given our finding that IL16 mediates recruitment of CD8+T cells in SLE, we suggest that IL16 could promote chemoattraction of CD8+T cells to inflammatory sites. Adding anti-IL16 antibodies attenuated transmigration of CD8+T cells in SLE samples.

Refined analysis of transmigration of CD4+T cell subsets revealed that in SLE, but not in controls, IL16 stimulation enhanced migration of Th1-like cells. Th1 cells are regarded as major players in SLE pathogenesis and are found in LN kidney infiltrates and urine [38–40]. Th1 cells are involved in immune attack of tissue cells, promoting tissue inflammation via secreted cytokines and facilitating LN progression [41]. We found reduced IL16 expression in circulating Th1-like cells in A-SLE, especially in LN, which was accompanied by higher levels of uIL16. Two independent reports demonstrated that a large proportion of kidney infiltrating cells are positive for IL16 [14, 16]. Altogether, available information raises the hypothesis that Th1 cells could be recruited by IL16 to the LN kidney infiltrates. Further, we found that Th1 migration could be attenuated by IL16 blockade.

Moreover, we sought to investigate which IL16 co-receptors were possibly expressed on Th1, CCR5 or CXCR4, and mediated cell recruitment. Earlier studies in rheumatoid arthritis showed increased numbers of Th1 cells in synovial fluid with upregulated CCR5 [42]. Studies of CCR5 expression in SLE are scarce, but high numbers of circulating CD4+CCR5+T cells associate with risk of atherosclerosis [43]. In our study, migrated Th1-like cells were enriched for CXCR4 and CCR5 and anti-IL16 treatment attenuated this effect, thus strongly suggesting that IL16 could mediate recruitment of Th1 cells via both receptors.

We and others have demonstrated the importance of Th17 responses in SLE [44]. In this study, we did not find differences in IL16+ Th17 cells between SLE patients and controls. Stimulation with IL16 did not affect proportions of migrated Th17 cells in either group. However, migrated cells from both patients and controls upregulated CD69 and HLA-DR, suggesting that Th17 cells may become activated upon IL16 exposure. Thus, while IL16 does not induce their migration, it may contribute to their activation.

The observations from the *in vitro* assays introduce the question of whether *in vivo* IL16 exerts the same effects and whether circulating and tissue resident immune cells behave similarly once exposed to the cytokine. In a broader perspective, the question relates both to how much circulating and tissue resident immune cells diverge in their phenotypical and functional features, and how autoimmune cells in peripheral tissues behave [40, 45]. The composition of the peripheral pool of immune cells varies in diseases such as SLE and associates with disease activity and clinical phenotype, and thereby is subjected to dynamic changes over time and, likely, by treatments [46]. Importantly we included active LN and active non-LN patients, and compared those with inactive patients in order to cover some of these aspects.

Although well characterized, our SLE cohort is still small which may limit discoverable associations and reduce statistical power. Additionally, patients received a variety of treatments, reflecting real-life clinical heterogeneity, which we were unable to fully control or stratify for in this study. For *in vitro* analyses, experiments were designed to provide insights into the role of IL16 in disease mechanisms, particularly by examining its roles in enhancing plasma cell differentiation and T cell migration. While these findings offer valuable concepts, further studies with larger sample sizes and more controlled conditions may help validate the observations and unravel more details of IL16 biology.

For the characterization of IL16 expression, the experiment was designed to determine its expression in major immune cell compartments. However, due to limitations in cell samples as well as in our flow cytometry panel, several interesting T cell subsets were not investigated (e.g. Th2, follicular and peripheral helper T cells). Further studies investigating IL16 expression in these subsets may provide deeper insight into IL16-mediated mechanisms in SLE.

In conclusion, we present unique data on functional roles of IL16 on immune cells in SLE. We demonstrate that SLE patients have reduced proportions of IL16+ cells among circulating immune cells, especially B, T, and NK cells, with further reduction of IL16+ CD4+T cells observed in LN. This correlates with increased uIL16, suggesting a putative role of secreted IL16 in LN, e.g. by promoting migration of CD4+Th1 and CD8+T cells. Our data contributes to understanding of IL16’s role in SLE, and particularly in LN and pave a path for tailoring strategies for IL16 blocking therapies in the future.

## Supplementary Material

uxaf068_Supplementary_Data

## Data Availability

The data generated during and/or analysed during the current study are available from the corresponding author on request and approval of ONO pharma.

## Abbreviations

A: Active
aNAV: activated naive
ACR: American College of Rheumatology
autoAbs: Autoantibodies
CCR5: C-C chemokine receptor 5
CXCR4: C-X-C chemokine receptor 4
C: complement
DN: double negative
eGFR: estimated glomerular filtration rate
HC: healthy control
HRP: horseradish peroxidase
IL: interleukin
IFL: indirect immunofluorescence method
I: inactive
KS: Karolinska University Hospital
LN: lupus nephritis
NK: natural killer
NKT: natural killer T
NAV: naive
PBMCs: peripheral blood mononuclear cells
P: plasma
PB: plasmablasts
Treg: regulatory T
SLE: systemic lupus erythematosus
SLICC: systemic lupus international collaborating clinics
SWM: switched memory
Th: T helper
TMB: tetramethylbenzidine enzyme substrate
USW: unswitched memory
US: unstimulated cells
U: urine
