# Supplementary material for: Interleukin 16 in lupus nephritis—a role for Th1 and CD8+ T cell migration
Source: Clin Exp Immunol. 2025 Oct 8;219(1):uxaf068. doi: 10.1093/cei/uxaf068 (PMC12969281; doi:10.1093/cei/uxaf068)
Supplement: uxaf068_Supplementary_Data [file uxaf068_supplementary_data.docx]

**Supplementary table 1 Flow cytometry panel used for this study**

| Antibody | Fluorophore | Clone | Company | Dilution | Catalogue No. |
| --- | --- | --- | --- | --- | --- |
| Fixable viability dye | Zombie NIR | - | Biolegend | 1:2000 | 423105 |
| IgG | BV421 | G18-145 | BD | 1:50 | 562581 |
| CD20 | PerCP | 2H7 | Biolegend | 1:20 | 302324 |
| CD27 | BUV496 | O323 | BD | 1:100 | 751678 |
| IgD | BV605 | IA6-2 | Biolegend | 1:50 | 348232 |
| CD38 | PE/CF594 | HIT-2 | BD | 1:100 | 562288 |
| CXCR5 | BUV563 | RF8B2 | BD | 1:50 | 741316 |
| IgM | BV570 | MHM-88 | Biolegend | 1:50 | 314518 |
| CD19 | AF532 | HIB19 | ThermoFisher | 1:25 | 58-0199-42 |
| CD56 | PE/Cy5 | B159 | BD | 1:100 | 555517 |
| CD9 | PE/Cy7 | HI9a | Biolegend | 1:50 | 312116 |
| CD21 | BUV737 | B-ly4 | BD | 1:50 | 612789 |
| CD11c | BUV805 | B-ly6 | BD | 1:50 | 742005 |
| HLA-DR | BV650 | L243 | Biolegend | 1:400 | 307650 |
| CD69 | BV750 | FN50 | BD | 1:100 | 747522 |
| CD95 | APC | DX2 | Biolegend | 1:100 | 305612 |
| CD3 | PE/Cy5.5 | 7D6 | ThermoFisher | 1:100 | MHCD0318 |
| CD4 | BV786 | SK3 | BD | 1:100 | 563877 |
| CD8 | BUV395 | RPA-T8 | BD | 1:100 | 563795 |
| CXCR3 | APC/Fire810 | G025H7 | Biolegend | 1:25 | 353762 |
| CCR6 | BUV661 | 11A9 | BD | 1:25 | 750696 |
| CCR7 | PE/Fire810 | G043H7 | Biolegend | 1:100 | 353269 |
| CD45Ra | AF700 | HI100 | Biolegend | 1:100 | 304120 |
| PD1 | SB436 | eBioJ105 | ThermoFisher | 1:50 | 62-2799-42 |
| TCRgd | BV711 | 11F2 | BD | 1:100 | 568490 |
| CD127 | PacificBlue | A019D5 | Biolegend | 1:50 | 351306 |
| CD25 | BV480 | BC96 | BD | 1:100 | 567488 |
| CXCR4 | BB700 | 12G5 | BD | 1:50 | 566553 |
| CCR5 | PerCP-eFluor710 | NP-6G4 | ThermoFishe | 1:25 | 46-1956-42 |
| CD14 | FITC | M5E2 | BD | 1:100 | 555397 |
| CD16 | APC/H7 | 3G8 | BD | 1:100 | 560195 |
| IL16 | PE | 14.1 | Biolegend | 1:100 | 519106 |

**Supplementary table 2 Antibodies used for CD4+T cell sorting**

| Antibody | Fluorophore | Clone | Company | Dilution | Catalogue No. |
| --- | --- | --- | --- | --- | --- |
| Fixable viability dye | Green | - | Biolegend | 1:200 | 423107 |
| CD3 | PE/Cy5.5 | 7D6 | ThermoFisher | 1:25 | MHCD0318 |
| CD4 | BV421 | RPA-T4 | BD | 1:25 | 562424 |
| CD8 | AF647 | RPA-T8 | BD | 1:25 | 557708 |
| CD14 | APC/H7 | M5E2 | BD | 1:25 | 561384 |
| CD16 | APC/H7 | 3G8 | BD | 1:25 | 560195 |
| CD19 | APC/H7 | SJ25C1 | BD | 1:25 | 560177 |

**Supplementary table 3 Characteristics of lupus nephritis and non-lupus nephritis patient**

| Categories | Features | LN patients | Non-LN patients |
| --- | --- | --- | --- |
| Demographic | No. of patients, No (%) | 16/32 (50%) | 16/32 (50%) |
|  | Age, years (#) | 38 (30-45.5) | 47.5 (37.5-56.5) |
|  | Gender |  |  |
|  | - Male, No (%) - Female, No (%) | 1/16 (6%)  15/16 (94%) | 1/16 (6%)  15/16 (94%) |
|  | SLEDAI-2K score | 12 (8-14) | 6 (4-9) |
|  | Disease duration, years (#) | 6.5 (4-20) | 16 (4.5-24.5) |
| Laboratory parameters | Plasma creatinine, µmol/L (#) | 67 (53-85.5) | 68 (57-75.5) |
|  | ESR rates, mm/h (#) | 23 (12-43) | 25 (12-61) |
|  | C1q levels, mg/L (#) | 124 (88-151) | 163 (98-246) |
|  | C3 levels, g/L (#) | 0.78 (0.64-1.01) | 0.82 (0.6-0.96) |
|  | C3d levels, g/L (#) | 8 (7-9) | 7.5 (6-9) |
|  | C4 levels, g/L (#) | 0.12 (0.09-0.2) | 0.08 (0.04-0.17) |
|  | Anti-dsDNA positive, N0 (#) | 10/16 (62%) | 9/16 (56%) |
| Treatment at inclusion | Mean dose (Prednisolone equivalent, mg) | 7.5 | 10 |
|  | Antimalarial | 13/16 (81%) | 10/16 (62%) |
| *sDMARDs* | Azathioprine | 0/16 (0%) | 0/16 (0%) |
|  | Mycophenolate mofetil | 6/16 (38%) | 2/16 (12%) |
|  | Calcineurin inhibitors | 2/16 (12%) | 0/16 (0%) |
| *bDMARDs* | Belimumab | 0/16 (0%) | 0/16 (0%) |
|  | Rituximab | 2/16 (12%) | 2/16 (12%) |

ND: Not done; SLE: Systemic Lupus Erythematosus; SLEDAI-2K: Systemic Lupus Erythematosus Disease Activity 2000; ESR: Erythrocyte Sedimentation Rate; C1q: Complement 1q; C3: Complement 3; C3d: Complement 3d; C4: Complement 4; Anti-dsDNA: Anti-double strand DNA; sDMARDs: synthetic disease-modifying antirheumatic drugs; bDMARDs: biologic disease-modifying antirheumatic drugs; # Median (Q1-Q3).

**Supplementary table 4 Characteristics of SLE patients used in soluble IL16 measurement**

| Categories | Features | SLE | |
| --- | --- | --- | --- |
|  |  | **Plasma** | **Urine** |
| Demographic | No. of LN patients, No (%) | 13/28 (46%) | 10/19 (52%) |
|  | Age, years (#) | 41 (30-56) | 42.5 (30-49) |
|  | Gender |  |  |
|  | - Male, No (%) - Female, No (%) | 2/28 (7%)  26/28 (93%) | 1/19 (5%)  18/19 (95%) |
|  | SLEDAI-2K score (#) | 8 (6-12) | 8 (6-12) |
|  | Disease duration, years (#) | 8.5 (4-20) | 7 (4-19) |
| Laboratory parameters | Plasma creatinine, µmol/L (#) | 68 (54.5-76) | 63 (51-76) |
|  | ESR rates, mm/h (#) | 21.5 (12.5-47.5) | 17 (9-47) |
|  | C1q levels, g/L (#) | 150 (101-240) | 146 (98-205) |
|  | C3 levels, g/L (#) | 0.82 (0.62-1.1) | 0.71 (0.58-1.1) |
|  | C3d levels, g/L (#) | 8 (5-9) | 8 (5-8) |
|  | C4 levels, g/L (#) | 0.11 (0.08-0.19) | 0.1 (0.05-0.24) |
|  | Anti-dsDNA positive, N0 (#) | 17/28 (61%) | 13/19 (68%) |
| Treatment at inclusion | Mean dose (Prednisolone equivalent, mg) | 7.5 | 7.5 |
|  | Antimalarial | 23/28 (82%) | 15/19 (78%) |
| *sDMARDs* | Azathioprine | 1/28 (4%) | 0/19 (0%) |
|  | Mycophenolate mofetil | 7/28 (25%) | 6/19 (31%) |
|  | Calcineurin inhibitors | 2/28 (7%) | 1/19 (5%) |
| *bDMARDs* | Belimumab | 0/28 (0%) | 0/19(0%) |
|  | Rituximab | 4/28 (14%) | 3/19 (15%) |

ND: Not done; SLE: Systemic Lupus Erythematosus; SLEDAI-2K: Systemic Lupus Erythematosus Disease Activity 2000; ESR: Erythrocyte Sedimentation Rate; C1q: Complement 1q; C3: Complement 3; C3d: Complement 3d; C4: Complement 4; Anti-dsDNA: Anti-double strand DNA; sDMARDs: synthetic disease-modifying antirheumatic drugs; bDMARDs: biologic disease-modifying antirheumatic drugs; # Median (Q1-Q3).

**Supplementary table 5 Characteristics of SLE patients and healthy controls used in *in Vitro* studies**

| Categories | Features | SLE | HC |
| --- | --- | --- | --- |
| Demographic | No. of LN patients, No (%) | 6/10 (60%) | 7 |
|  | Age, years (#) | 37 (30-47) | 37.5 (31-51) |
|  | Gender |  |  |
|  | - Male, No (%) - Female, No (%) | 0/10 (0%)  10/10 (100%) | 0/7 (0%)  7/7 (100%) |
|  | SLEDAI-2K score | 8.5 (6-12) | ND |
|  | Disease duration, years (#) | 12 (2-23) | ND |
| Laboratory parameters | Plasma creatinine, µmol/L (#) | 72 (55-96) | ND |
|  | ESR rates, mm/h (#) | 43.5 (23-54.5) | ND |
|  | C1q levels, mg/L (#) | 152.5 (114-205) | ND |
|  | C3 levels, g/L (#) | 0.66 (0.6-1.18) | ND |
|  | C3d levels, g/L (#) | 10.5 (8-13) | ND |
|  | C4 levels, g/L (#) | 0.07 (0.04-0.24) | ND |
|  | Anti-dsDNA positive, N0 (#) | 8/10 (80%) | ND |
| Treatment at inclusion | Mean dose (Prednisolone equivalent, mg) | 10 | ND |
|  | Antimalarial | 9/10 (90%) | ND |
| *sDMARDs* | Azathioprine | 2/10 (20%) | ND |
|  | Mycophenolate mofetil | 4/10 (40%) | ND |
|  | Calcineurin inhibitors | 2/10 (20%) | ND |
| *bDMARDs* | Belimumab | 0/10 (0%) | ND |
|  | Rituximab | 0/10 (0%) | ND |

ND: Not done; SLE: Systemic Lupus Erythematosus; SLEDAI-2K: Systemic Lupus Erythematosus Disease Activity 2000; ESR: Erythrocyte Sedimentation Rate; C1q: Complement 1q; C3: Complement 3; C3d: Complement 3d; C4: Complement 4; Anti-dsDNA: Anti-double strand DNA; sDMARDs: synthetic disease-modifying antirheumatic drugs; bDMARDs: biologic disease-modifying antirheumatic drugs; # Median (Q1-Q3).

**Supplementary figure 1 Study design.** The schematic demonstration of workflow of the cohort. Continuous line indicates samples that were taken for further experiments. A-SLE: active SLE, I-SLE: inactive SLE, ELISA: enzyme-linked immunosorbent assay, PBMCs: peripheral blood mononuclear cells, LN: lupus nephritis.

**Supplementary figure 2 Representation of gating strategy of lymphocyte subpopulations in SLE patients.** (A) Different immune cell compartments dissection including CD4+T (CD3^+^CD8^-^CD4^+^), CD8+T (CD3^+^CD4^-^CD8^+^), B (CD3^-^CD19^+^), natural killer (NK: CD3^-^CD56^+^) and natural killer T (NKT: CD3^+^CD56^+^). (B) Different B cell subsets including plasmablasts (PB: CD27^hi^CD38^hi^), switched memory (SWM: CD27^+^IgD^-^), unswitched memory (USW: CD27^+^IgD^+^), double negative (DN: CD27^-^IgD^-^) and naive (NAV: CD27^-^IgD^+^) B cells. (C) Different CD4+T cell subsets including T helper 1 (Th1: CXCR3^+^CCR6^-^), T helper 17 (Th17: CXCR3^-^CCR6^+^), non Th1/Th17 (CXCR3^-^CCR6^-^) and regulatory T (Treg: CD127^-^CD25^+^) cells.

**Supplementary figure 3 Histograms represent flow cytometry results from intracellular IL16 expression in different immune cell subsets.** (A) The grey and non-filled histograms show the intracellular IL16 staining and fluorescent minus one (FMO) of IL16. (B) Flow cytometric plots represent IL16 expression among different lymphocyte subsets in patients with active, inactive and HC.

**Supplementary figure 4** Violin plots represented IL16 expression in all cell clusters, macrophages (CM0-CM4), T and NK (CT0-CT6), and B cells (CB0-CB3), in patients with lupus nephritis. The figure was generated by website (https://singlecell.broadinstitute.org/single_cell/study/SCP279/amp-phase-1#study-summary).

**Supplementary figure 5 Frequency of IL16-positive cells among double negative and naïve B cell subsets.** Frequency of IL16+ cells in double negative 1 (DN1: CD19+CD27-IgD-CD21+CD11c-), double negative 2 (DN2: CD19+CD27-IgD-CD21-CD11c+), double negative 3 (DN3: CD19+CD27-IgD-CD21-CD11c-), resting naive (rNAV: CD19+CD27-IgD+CD21+CD11c-) and activated naive (aNAV: CD19+CD27-IgD+CD21-CD11c+) in (A) HC, inactive and active patients, and (B) LN and non-LN patients.

**Supplementary figure 6.** (A) Correlation analysis of plasma (p) and urine (u) IL16 levels in SLE and LN patients. (B) Longitudinal changes in pIL16 and uIL16 levels at baseline and follow-up time points (3 and 6 months) in LN and non-LN patients. Disease activity (SLEDAI-2K) and complement 4 (C4) levels in patients (C) who had high- and low-levels of plasma IL16 and (D) who had high- and low-levels of urine IL16.

**Supplementary figure 7.** (A) Gating strategies represent plasma cells (CD19+CD27+CD38+) of patients after stimulation with four different stimulating conditions, including S1 (R848 and IL2), S2 (R848, IL2 and IL16), S3 (R848, IL2 and IL21) and S4 (R848, IL2, IL16 and IL21). (B) Frequency of plasma cells and levels of (C) immunoglobulin G (IgG) and M (IgM) after stimulation. (D) Histograms show the expression of HLA-DR and CD95 of plasma cells after stimulation. (E) MFI of HLA-DR and CD95 expression on plasma cells after stimulation.

**Supplementary figure 8.** (A) Frequencies of migratory lymphocytes among HC and A-SLE after stimulation of CCL5 and IL16 (at 10, 10^2^, 10^3^, 10^4^ pg/ml), and unstimulated condition. (B) MFI of CCR5 expression in migratory CD8+T cells under different stimulation conditions (C) Frequency of lupus transmigrated-CD4+T cells after addition of anti-human IL16 in different stimulated-IL16 concentrations (at 10, 10^2^, 10^3^, 10^4^ pg/ml). Frequencies of (D) non-migratory Th1/Th17 cell in SLE patients and HC under different stimulation conditions, and (E) of lupus transmigrated-non Th1/Th17 cells after addition of anti-human IL16 in different stimulated-IL16 concentrations (at 10, 10^2^, 10^3^, 10^4^ pg/ml).
